# On a Cause of Jaundice

**Published:** 1801-10

**Authors:** G. Fletcher

**Affiliations:** Chesterfield


### *On a Cause of Jaundice.*

To the EDITORS of the MEDICAL and PHYSICAL JOURNAL.

Gentlemen,

IN Number XXIX of your valuable Publication, Dr. Crichton has given an account of " an undefcribed caufe of Jaundice," which was a fcirrhous pancreas, and upon which Mr. Bayley obferves, in your laft Number, that fuch a caufe of jaundice was publifhed by him in 1799. My reafon for troubling you with this letter is merely to point out that this caufe of jaundice has been long fince defcribed by Chefelden in his Anatomy. His edition which I have is the 7th octavo edition, publifhed at London in 1750; and at p. 166 are thefe words: " In a man that died of a jaundice, I found the ductus communis choledochus *constricted by a scirrhous pancreas*, the gall bladder extended to the fize of a goofe egg, and all the ducts to twice their natural bignefs." This is almoft an exact defcription of the firft cafe mentioned by Dr. Crichton, where, upon diffection, he found the ductus communis furrounded by the extremity of a fcirrhous pancreas. Profeffor Harwood, of Cambridge, mentions, in his Anatomical Lectures, a cafe of a boy who died in the County Infirmary, of a jaundice, and, " upon opening the body, he found the caufe to be a *scirrhous pancreas, which pressed on the ductus communis* fo as to form a complete obftruction to the paffage of the bile, and thofe parts in which the bile were detained were fwelled to a moft aftonifhing fize. This difeafe was brought on by a kick from a horfe." This is an extract from my notes of the Profeffor's Lectures, taken in the year 1788. When I firft read Dr. Crichton's account, I fuppofed by an undefcribed caufe of jaundice, he meant a caufe not defcribed in any book of Nofology; but Mr. Bayley underftands him differently. If Dr. C. thought that a fcirrhous pancreas as a caufe of jaundice had not been defcribed and publifhed, he certainly was miftaken; and it is for the purpofe of pointing out this fimple fact, and not with a view of detracting from the utility of his obfervations upon it, that I have fent you the preceding quotation from Chefelden.

I am, &c.

